# SH2 Domains: Folding, Binding and Therapeutical Approaches

**DOI:** 10.3390/ijms232415944

**Published:** 2022-12-15

**Authors:** Awa Diop, Daniele Santorelli, Francesca Malagrinò, Caterina Nardella, Valeria Pennacchietti, Livia Pagano, Lucia Marcocci, Paola Pietrangeli, Stefano Gianni, Angelo Toto

**Affiliations:** Istituto Pasteur—Fondazione Cenci Bolognetti, Dipartimento di Scienze Biochimiche “A. Rossi Fanelli” and Istituto di Biologia e Patologia Molecolari del CNR, Sapienza Università di Roma, 00185 Rome, Italy

**Keywords:** protein-protein interactions, phosphotyrosine, Src Homology 2

## Abstract

SH2 (Src Homology 2) domains are among the best characterized and most studied protein-protein interaction (PPIs) modules able to bind and recognize sequences presenting a phosphorylated tyrosine. This post-translational modification is a key regulator of a plethora of physiological and molecular pathways in the eukaryotic cell, so SH2 domains possess a fundamental role in cell signaling. Consequently, several pathologies arise from the dysregulation of such SH2-domains mediated PPIs. In this review, we recapitulate the current knowledge about the structural, folding stability, and binding properties of SH2 domains and their roles in molecular pathways and pathogenesis. Moreover, we focus attention on the different strategies employed to modulate/inhibit SH2 domains binding. Altogether, the information gathered points to evidence that pharmacological interest in SH2 domains is highly strategic to developing new therapeutics. Moreover, a deeper understanding of the molecular determinants of the thermodynamic stability as well as of the binding properties of SH2 domains appears to be fundamental in order to improve the possibility of preventing their dysregulated interactions.

## 1. SH2 Domains Are the Archetypical “Readers” of Phosphorylated Tyrosines

Tyrosine phosphorylation is a key post-translational protein modification, with the main function of transducing signals in response to internal or external stimuli [1,2]. Tyrosine phosphorylation occurs mainly through the activity of specialized enzymes, Protein Tyrosine Kinases (PTKs), and it is at the basis of the regulation of a plethora of molecular and physiological cellular pathways, ranging from cell proliferation to differentiation. While PTKs act as “writer” enzymes and catalyze the transfer of a phosphate group from ATP to the substrate, another class of enzymes, Protein Tyrosine Phosphatases (PTPs), act as “erasers” and catalyzes the removal of the phosphate group from a defined tyrosine residue. The cellular activity of PTKs and PTPs is highly regulated and often operates as on-and-off switches activating and/or repressing specific molecular pathways.

In order to link PTKs to downstream signaling proteins and molecules, the presence of protein modules able to recognize and bind phosphorylated tyrosine residues is necessary. Among them, the SH2 (Src Homology 2) domain is one of the best characterized and studied. Other examples of domains that can interact with phosphotyrosine are the PTB (PhosphoTyrosine Binding) domain [3], the C2 domain [4], and the HYB domain [5]. Given their abundance in the human proteome (111 proteins are known to possess at least one SH2 domain in their sequence, for a total of 121 SH2 domains [6]; a list of SH2-containing proteins is reported in Table 1) and their key role in cell signaling, SH2 domains are involved in several human pathologies, ranging from genetic disorders affecting the normal development of tissue and organs [7] to several kinds of diseases and cancer [8,9,10,11]. Since protein-protein interactions are strictly regulated in space and time in the cellular environment, mutations affecting the ability to bind a specific ligand, affecting the thermodynamic stability of the complex and/or the kinetic properties of the binding event, may lead to dysregulation of key molecular and physiological pathways.

In this review article, we analyze the current knowledge about the structural, folding stability, and binding properties of SH2 domains, their roles in the onset of pathological conditions, and the different strategies designed to modulate/inhibit the function of SH2 domains. Our analysis highlights that SH2 domains represent a key target of pharmacological interest for developing new therapeutic strategies. For this reason, a deeper understanding of the molecular determinants of thermodynamic stability as well as of the binding properties of SH2 domains is needed to increase our ability to prevent their dysregulated interactions.

## 2. Folding Properties of SH2 Domains

SH2 domains are key mediators of the recognition of phosphorylated tyrosines, with the consequent prominent involvement in regulating a plethora of molecular and physiological pathways; however, only a few experimental works characterized the determinants of the thermodynamic stability and the folding of SH2 domains. Comprehending these aspects is fundamental in order to depict the molecular basis of the function of SH2 domains.

The resolution of a high number of SH2 domain three-dimensional structures highlights a highly conserved topology among the domain family [12,13]. They are ~100 residue globular domains, structurally arranged as two α-helices flanking three to five anti-parallel β strands that compose a central β sheet. This highly conserved three-dimensional structure makes SH2 domains perfect model systems for folding studies. In fact, one powerful methodology that allows one to understand the molecular details of the folding mechanism of a given protein is to compare its folding properties with other proteins sharing the same structure but possessing different amino acid sequences.

The folding pathway of the N-SH2 and C-SH2 domains of SHP2 has been characterized in great detail. The analysis of the (un)folding kinetics of N-SH2 revealed the presence of an intermediate along the reaction pathway. In fact, while the typical chevron plots (i.e., a semilogarithmic plot of the observed unfolding and refolding rate constant as a function of the concentration of denaturant) for two-state folding proteins is V-shaped [14], the N-SH2 domain chevron-plot reported a clear curvature in the refolding arm (the so-called roll-over effect) which represents a clear sign of the presence of an intermediate along the reaction [15]. Quantitative analysis of kinetic data allowed us to conclude that the intermediate is a low-energy species transiently populated during the folding reaction [16]. On the other hand, the folding kinetics of the C-SH2 domains obtained at a wide range of experimental conditions were different compared to the N-SH2 domain. In fact, the analysis of the chevron plot revealed a roll-over effect in the unfolding arm, and a rigorous analysis of the kinetic parameters revealed a folding scenario implying the presence of two transition states and a high-energy, never accumulating intermediate.

**Figure 1:**
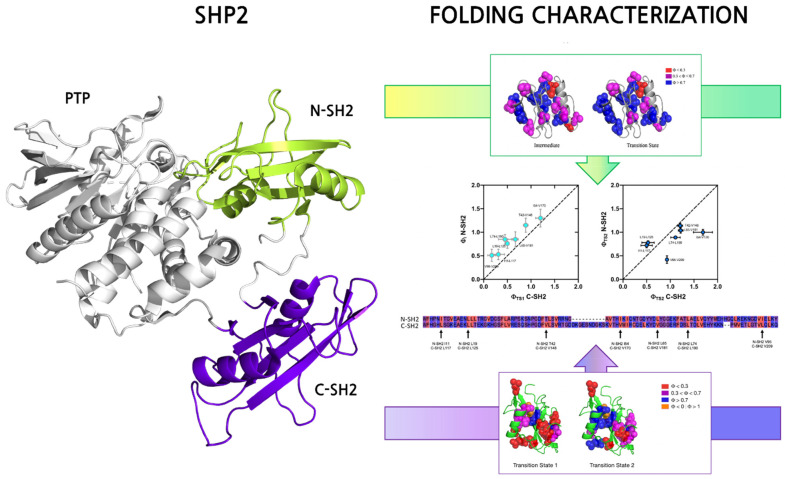
**(Left**) structure of Shp2 protein composed of two contiguous SH2 domains (N- and C-SH2, in green and purple, respectively) and a PTP catalytic domain. PDBcode: 2shp. (**Right**) Folding characterization of N-SH2 (reproduced with permission from [19]) and C-SH2 (reproduced under the terms of the Creative Commons Attribution License from [20]) domains of Shp2. The ϕ value analysis carried out using the isolated domains allows for defining the native-like interactions occurring in the transition state(s) of the folding reaction. Data obtained were then used to obtain a Φ vs. Φ plots (in the center) of early (top left panel) and late (top right panel) events of the folding reaction of N-SH2 versus C-SH2 domain (see references and text for details).

A useful approach to obtain information about the folding mechanism of a given domain is to gather structural details of the transition state(s) and intermediate(s) that occur along the reaction pathway. However, given their typical elusiveness to an experimental characterization, a structural characterization of transition states and intermediates can be performed only indirectly. A powerful methodology that can be applied to obtain such information is the ϕ-value analysis, an approach that relies on extensively mutating individual residues of the domain and monitoring the effect of mutations on the folding kinetics, inferring the role of each residue in the folding reaction and normalizing it in terms of native-like interactions in the probed state (details about the ϕ-value analysis methodology can be found at [17,18]). Both N-SH2 and C-SH2 were subjected to a ϕ-value analysis [19,20] (Figure 1). The analysis of ϕ-values obtained for the N-SH2 domain revealed that the intermediate state (early event of folding) is highly structured, with native-like interactions further locked in place in the late transition state. Interestingly, a comparison with the ϕ-values obtained for the C-SH2 highlights that the latter experiences a much lower degree of native-like interactions in the early events of folding. This aspect suggests that the two domains that possess an almost perfectly superimposable three-dimensional structure may be characterized by a different degree of residual structure in the denatured state, which influences and dictates the early events of folding. However, quantitative analysis of kinetic data highlights a generally conserved folding mechanism between the C-SH2 and N-SH2 domains.

Other SH2 domains that have been characterized in their folding properties are the SH2 domain of Src [21], the N-terminal and C-terminal SH2 domains of the p85 subunit of PI3K [22,23], and the SH2 domain of Crkl [24]. It is interesting that the SH2 domain from Crkl displays similar folding kinetics compared to the N-SH2 domain of SHP2, with a pronounced roll-over effect in the refolding arm of the chevron plot being compatible with an energetic profile implying the presence of an obligatory intermediate accumulating along the reaction. A peculiar case is represented by the C-SH2 domain of PI3KR (p85 regulatory subunit of PI3K). In this case, the analysis of kinetic data revealed that the domain populates a low-energy intermediate determined by peptidyl-prolyl cis-trans isomerization [23].

It would be tempting to speculate that the presence of intermediate(s) is mandatory for the productive folding of this protein family. However, the folding pathway of the C-SH2 domain of SHP2, which is characterized by a high-energy not accumulating intermediate, and experimental data obtained for the N-SH2 domain of p85 and the SH2 domain of Src that are compatible with a simple two-state folding mechanism [21,22,25] suggest that the presence of multiple energy minima is not obligatory for the folding SH2 domains. Further experimental data based on extensive mutagenesis, as well as on multidomain folding, are crucial for a better understanding of the folding properties of SH2 domains. In fact, since topology and function are tightly correlated in globular domains, deciphering the mechanism by how SH2 domains acquire their three-dimensional structure may unveil new perspectives on the determinants of their function. Moreover, mutations altering the function of the SH2 domain may not occur in the binding pocket (i.e., directly affecting protein-protein interaction) and may result in populating misfolded species that could disrupt SH2 domain function.

## 3. SH2 Domain Binding Properties

In general, two main events characterize protein-protein interactions, (i) recognition of a specific consensus sequence and (ii) stabilization of the bound complex, whose determinants can vary throughout domain families [26,27,28,29]. For SH2 domains, these aspects are crucial, given their prominent role in mediating the binding of ligands presenting a single specific post-translationally modified residue, binding specificity must occur by the recognition of different consensus sequences flanking the phosphotyrosine [1,30,31]. During the co-evolution with their ligands, members of the SH2 domain family adopted different strategies to specifically recognize the target sequences carrying pTyr residues, even though they preserved the same fold [32]. As observed for mostprotein families, the loops connecting secondary structural elements of SH2 domains are the most flexible and dynamic regions. In addition, recent works have shown that the wide spectrum of specificity observed in SH2 domains can depend on the loop variation [33]. However, the discovery that loops play key roles in the selectivity of SH2 domains not only provides a glimpse of the molecular basis of the SH2 domain/phosphopeptide recognition but also opens new frontiers at the development of specific inhibitors targeting interactions of SH2 domains implicated in pathological conditions [34].

### 3.1. Defining the Structural Determinants of Recognition and Specificity

SH2 domains are considered key components of the pTyr-dependent signal transduction networks in eukaryotic cells [35], which are triggered by a wide range of external and internal stimuli. In this scenario, SH2 domains mediate the binding events through a fine regulation necessary for proper signaling and rapid cellular response [34,36,37,38]. 

**Figure 2:**
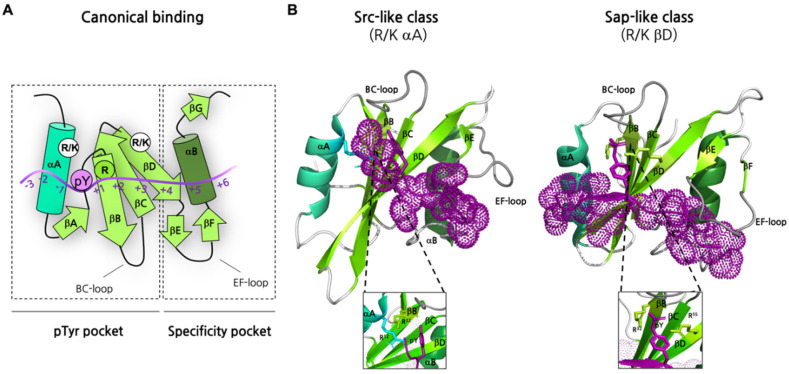
(**A**) Scheme of a canonical binding reaction between an SH2 domain (in green) and a Tyrosine-phosphorylated peptide (in violet). The peptide is numbered by counting pTyr as 0. In detail, we can distinguish two structural binding elements in the SH2 domain; pTyr pocket—composed by helix αA (in cyano), beta-strands βB, βC, and βD (in light green), and the BC-loop—and a specificity pocket—composed by helix αB (dark green), beta-strands βD, βE, βF and βG (light green) BG loop and the EF loop. Arg^32^ in the pTyr pocket (green circle) is a highly conserved residue driving the interaction in all SH2 domains. Arg/Lys on αA or βD (white circles) can assist the reaction. (**B**) Examples of interactions between SH2 domains and pTyr-peptides mediated by Arg32 and an Arg residue on αA in case of Src-like class of binding (left, PDBcode:1sps), and an Arg residue on βD in case of Sap-like class of binding (right, PDBcode:1d4w). Structural details in the squares. The conserved binding motif is highlighted in blue. The motif corresponds to FLVR and YLVR in reported examples of Src-like (canonical) and Sap-like (non-canonical) classes of binding, respectively.

SH2 domains recognize and bind sequences of ~10 residues, generally referred to as Short Linear Motifs (SLiMs) [39,40]. SLiMs are generally intrinsically disordered, i.e., they do not possess a well-defined three-dimensional structure and are highly dynamic in solution, although they can acquire structure upon binding with their interactor(s) [41,42]. SLiMs recognized by the SH2 domain can be found in structured or disordered protein regions, although disordered regions have been proposed to possess higher biological and physiological significance [43]. In SH2 domains, two structural regions determine the protein-ligand interaction [44]: (i) the pTyr binding site, a groove formed by αA helix, βB/βC/βD strands, and the BC loop, and (ii) the specificity pocket, which is a relatively large hydrophobic pocket mainly delimited by residues of αB helix, βD strand, and BG and EF loops (Figure 2A). The specificity pocket accommodates residues that are C-terminal to the pTyr. It is of interest to notice that the alignment of almost one hundred SH2 domain structures present in the Protein Data Bank (PDB) revealed that the N-terminal region that provides a pTyr-binding pocket is more conserved than the C-terminal half of the SH2 domain, which instead exhibits greater structural variability [35]. In fact, most conserved residues are clustered on the βB strand, where a conserved arginine residue in the FLVR motif (Figure 2B) (Arg βB5 or Arg175 in the v-Src SH2 domain) plays the central role in forming a double hydrogen bond with the phosphate group of pTyr [45,46]. The binding affinities of the SH2 domain for the phosphotyrosine peptide are reported to be in the range of what is typically observed for protein-protein interactions [26], ranging from 10^−5^ to 10^−8^ M [16,24,25,47].

Additional residues that are key for phosphopeptide binding are His βD4, Lys βD6, and Arg αA2, which coordinate and anchor the aromatic ring of the phospho-tyrosine [48]. Recent studies have confirmed the importance of the protonation state of His in position βD4 in determining the change in binding affinity over pH. In particular, the His169 of the C-terminal SH2 domain of SHP2 protein and the His60 of CrkL protein is crucial for the binding properties of those SH2 domains. Moreover, thermodynamic studies have established that the ionic interaction between Arg βB5 and pTyr is crucial for the affinity, providing more than half of the total binding energy [49]. Accordingly, our previous kinetic studies have shown a strong dependence on the ionic strength of the aforementioned interaction [16,24,25]. The pioneering work of Cantley and co-workers on the phosphatidylinositol 3-kinase evidenced the key role of residues flanking the phosphotyrosine in determining the specificity of a ligand to a particular SH2 domain [50]. A few years later, another work predicted the binding motifs of twenty-five SH2 domains, first describing the sequence-based and structure-based regulation of the binding [51]. A more recent study defined the determinants of specificity of around two-thirds of the human SH2 domains using the OPAL approach (Oriented Peptide Array Library, a high throughput method to study specificities of protein domains [52]) and identified sequence motifs recognized by different SH2 domains [53]. This work established that most SH2 domains display binding preferences for specific residues at the +2, +3, and +4 positions (relative to pTyr) in the ligand sequence. It is possible to divide SH2 into three different groups based on the consensus sequence recognized, as summarized in Table 2.

**Figure 3:**
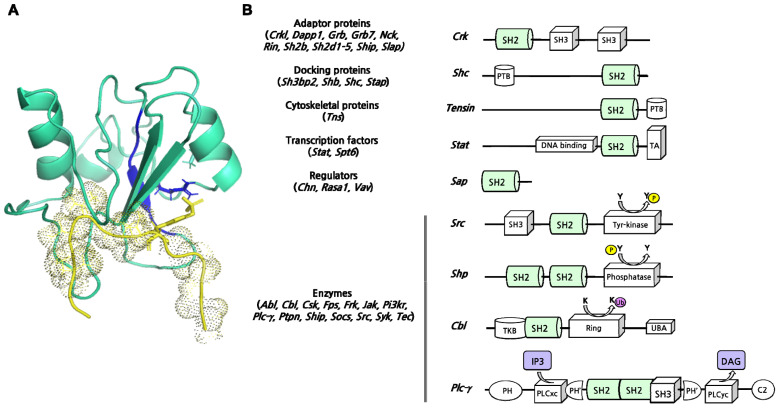
(**A**) A representative example of an SH2 domain in complex with a peptide mimicking a physiological ligand (PDBcode 1TCE). In detail, the SH2 domain of Shc (in light cyan) can interact with a tyrosine-phosphorylated peptide from the T cell receptor (in yellow) through a highly conserved sequence FLVRES (highlighted in blue). Arg32 (blue stick) is crucial for the interaction with the pY (yellow stick) harbored by the ligand. (**B**) Schematic representation of different SH2-domain-containing proteins exerting different molecular functions. In brackets, all protein families split according to the function (see Table 1 for references).

Similarly to what is observed for other protein-protein interaction domains, SH2 domains can be isolated or in tandem with other protein binding modules (Figure 3B). Many SH2 domain three-dimensional structures, isolated or in complex with their ligands, have been determined by X-ray and solution NMR (a representative example of the SH2 domain in complex with a peptide mimicking a physiological ligand is reported in Figure 3A), providing a fundamental contribution to the knowledge of the general aspects of the SH2-ligand recognition. The analysis of 63 human SH2 domains highlighted the simultaneous presence of the three binding pockets, which show selectivity for the +2, +3, and +4 residues of the phosphopeptide in all SH2 structures considered [33]. This observation led to hypothesizing a pivotal role of the SH2 domain loops in the control of the accessibility and shape of the binding pockets [33,54]. In particular, the study performed by Huang et al. highlighted that many SH2 domains exhibit specificity for a hydrophobic residue at the +3 position [53]. The nature of residue βD5 is crucial in determining this preference, whereas the +3 binding pocket is shaped by BG and EF loops [12,55]. Differently, 20 SH2 domains, including SH2 Grb2, displayed a preference for an asparagine residue at the +2 site of the ligand [53]. Consistent with the loops hypothesis, the structure of SH2 Grb2 in complex with its target sequence revealed that a tryptophan residue of the EF loop (TrpEF1) occupies the +3 binding pocket, favoring the interaction between Asn+2 of the peptide with βD6 and βE4 residues of the SH2 domain [56,57]. On the other side, SH2 domains listed in the group IIC were reported to be selective for a hydrophobic residue (preferentially Leu or Ile) at +4 position that is accommodated in a pocket formed by five hydrophobic residues (called “pentagon basket”). Interestingly, a Leu or Ile residue of the BG loop forms an intramolecular interaction inside the +4 binding pocket of those SH2 domains, not showing a preference for the +4 residue of the peptide [33,53]. In light of these structural considerations, the hypothesis of EF and BG loops as regulators of SH2 binding pockets has been supported by the engineering of new loops. In fact, by changing the loop sequence and conformation selectivity was altered as expected [33]. It is important to notice that the use of surface loops to determine binding specificity is not a distinct feature of SH2 domains [58,59,60,61,62]. On the other hand, the mechanism by which loops regulate binding pocket accessibility seems to be unique among protein-protein interaction modules [33].

### 3.2. Biophysical Characterization of SH2 Binding

Although SH2 domains are characterized by highly conserved fold and binding site structure, the molecular basis of selectivity is not completely understood, demanding additional experimental data aimed at characterizing from a thermodynamic and kinetic perspective the interaction occurring between SH2 domains and their ligands. Isothermal Titration Calorimetry (ITC) experiments have been carried out to study the affinity and selectivity of the SH2 domain of Src kinase [49]. A mutational analysis conducted on a peptide binding to the Src SH2 domain revealed that whilst conservative substitutions at +1, +2, and +3 positions resulted in minor destabilization, more drastic mutation to Ala revealed the +3 position as the most contributing to binding free energy. On the other hand, an analysis of the enthalpic change upon mutation showed the positions +1 and +2 to have a prominent role in providing an enthalpic contribution to binding, characterized by the formation of a highly ordered water molecule network [49]. These results provide useful information about the thermodynamics of the reaction, in particular by identifying entropic and enthalpic contribuitions to the change in free energy of the complex. However, equilibrium binding approaches are not feasible for a full understanding of the mechanism underlying the early recognition and the late stabilization events, for which kinetic approaches must be employed. 

Stopped-flow fast kinetic binding experiments allowed for measuring the microscopic association and dissociation rate constants (*k_on_* and *k_off_*, respectively) for the binding reaction of the SH2 domains from SHP2, PI3K, and CrkL proteins with peptides mimicking their physiological ligands [16,24,25,63,64]. Overall, kinetic data confirmed the importance of the electrostatic interactions in the early recognition events, as shown by the decrease of *k_on_* values at increasing ionic strength conditions. Moreover, conservative site-directed variants of N-terminal SH2 domains of SHP2 and PI3K were generated and used to determine the energetic contribution of mutated residues to the binding reaction with Gab2_608–620_ and Gab2_448–460_, respectively [63,64]. 

Results from the N-SH2 domain of the SHP2 protein highlighted five mutations (T42S, T52S, I56V, L65A, and L88A) that positively or negatively affected the affinity for Gab2_608–620_ by influencing the complex stability (as indicated by changes of *k_off_* values) rather than the early molecular recognition [63]. The mutation T42S caused a ten-fold destabilization of the complex, with a clear effect on the *k_off_* of the reaction, suggesting a prominent role of T42 in the late events of binding. Importantly, a more pronounced effect on the complex stability was also measured for the binding reaction involving the Noonan Syndrome-causing T42A variant of N-SH2 SHP2. In particular, for this reaction, the *k_off_* value resulted 100 times lower than what was measured for the interaction of wild-type N-SH2 with Gab2_608–620_ [65]. 

A mutational analysis conducted on the N-SH2 of PI3K showed that the affinity of the T48S variant for Gab2_448–460_ was around 20-fold lower than that of the wild-type form [64]. Interestingly, the T48 residue of N-SH2 PI3K and the T42 residue of N-SH2 SHP2 share the same position in the corresponding 3D structures [PDBs: 4QSY and 2IUH]. However, both *k_on_* and *k_off_* values of the binding reaction of N-SH2 PI3K with Gab2 were altered upon mutation T48, meaning that the threonine at this position is involved in both formation and stability of the complex. Further mutations of N-SH2 PI3K influencing the *k_on_* and/or *k_off_* values of the binding reaction with Gab2 peptide were identified. Interestingly, although affecting the kinetics of the reaction, those mutations did not necessarily determine a change of affinity for the ligand. In addition, the energetic coupling between the P74 residue and +3 methionine of the Gab2 peptide was highlighted, suggesting a role for this residue in determining the recognition of non-pTyr residue. Further kinetic experimental data and structural characterization of complexes are demanded to fully understand the determinants of specificity and affinity that allow SH2 domains to discriminate among different pTyr sites in the crowded cellular environment.

### 3.3. Non-Canonical SH2 Binding

Although it has been well accepted that selectivity relies on the molecular interaction of residues forming the specificity pocket of the SH2 domain with C-terminal residues to the pTyr, different works highlighted unusual pTyr binding modes increasing the spectrum of specificity observed in the SH2 domain family [66] (for which a graphical example is reported in Figure 2B, right panel). For instance, the Arg βB5 residue of the conserved motif FLVR is involved in the formation of a salt bridge with an aspartic acid residue in the C-terminal SH2 domain of p120RasGAP. Consequently, the pTyr recognition is mediated by other residues, such as the basic residues at βD4 and βD6 position, and residues in the BC loop, through a multi-dentate mechanism [67]. On the other hand, in some SH2 domains, Arg βB5 is not present, and the crucial binding of pTyr to this residue cannot occur. In fact, the FLVR arginine is replaced by histidine in the SH2 domain of RIN2 [38] and TYK2 [68] and tryptophan in SH2D5 [38].

TYK2 belongs to the JAK protein family, whose members contain SH2 domains that display a non-canonical binding mode. The JAK proteins are cytoplasmatic multi-domain tyrosine kinases that are associated with the intracellular domains of the cytokine receptors [69]. SH2 domains of JAK proteins specifically bind a glutamate residue instead of the pTyr, supporting the constitutive interaction of the JAK proteins with the cytokine receptors [68,70]. The lack of pTyr recognition caused the Arg residue typically conserved in SH2 domains to be replaced by a His in TYK2 (H474) [68]. Although TYK2 maintains the conserved binding pocket of SH2 domains, the H474 residue does not participate in the interaction with the ligand Ifnar1. The carboxylate group of E497 of Ifnar1 is coordinated by other residues of the binding pocket (S476 and T477). Moreover, the C-terminal hydrophobic residues of the target sequence pack into the hydrophobic groove formed between the EF loop and the βG1/2 hairpin of the SH2 TYK2, resembling the specificity pocket of the typical SH2 domain [68,69]. Another unconventional SH2 domain belongs to the SLAM-associated protein (SAP) (Figure 2B). Its binding pocket is uncommonly elongated and interacts with pY -3 and pY +2 residues of the target motif, forming a “three-pronged plug” interaction [71]. Surprisingly, SH2 SAP also recognizes non-phosphorylated motifs due to the additional binding site, but with a lower affinity compared to the sequences containing pTyr residues [11]. 

Other factors contributing to binding diversity in SH2 domains are represented by: (i) their ability to bind more than one phosphorylated site from the same ligand, (ii) their incorporation in a multidomain system where each SH2 domain interacts with different phosphorylated sites, (iii) their assembly depending by the oligomeric state of the protein of which they are part of [66]. For example, two phosphorylated sites of Syk are recognized by the SH2 domain of VAV or PLCγ1 [72,73]. Curiously, whilst the first pTyr site is recognized by following the classical “two-pronged plug” mechanism that involves the ArgβB5 residue, the second pTyr motif of Syk interacts with basic residues of the βD strand and BG loop of the SH2 domain. An example of a protein containing tandem SH2 domains is the ZAP-70 tyrosine-kinase. This SH2 tandem recognizes two pTyr sites of the ITAM cytoplasmatic tails. In particular, the C-terminal SH2 binds one pTyr site in a canonical “bidentate” way, whereas the N-terminal SH2 uses the ArgβB5 residue to coordinate the other pTyr and additional residues coming from C-SH2 to form the second dock site of its binding motif [74]. Moreover, both binding affinity and specificity can be influenced by the oligomeric state of the protein incorporating SH2 domains. This has been revealed in the adaptor protein APS, where the SH2 domain forms a dimer capable of binding four pTyr residues of insulin receptor tyrosine kinase. Interestingly, in the dimer, the canonical specificity pocket of one SH2 domain is interrupted by the long αB helix of the other SH2 domain; thus, the interaction with the ligand is mediated only by the pTyr residues recognition [75].

### 3.4. Biomolecular Condensate Formation Mediated by SH2 Domains

It has been recently discovered that the interactions between proteins do not form only simple protein-ligand complexes but can lead to the formation of highly ordered ensembles characterized by the formation of liquid droplet biomolecular clusters (or condensates) that regulate the physiological activity of the proteins involved [76]. The general properties of these protein condensates and the mechanisms by which they form are beyond the scope of this paper and have been diffusely reviewed elsewhere (see, for example, [77,78,79,80]). What appears clear is that the functions of protein interaction modules are highly dependent on the cellular context [81], so different mechanisms of ligand binding may follow changes in the intracellular microenvironment.

One of the fundamental aspects of the formation of such condensates is their correlation to post-translational modifications [82,83], which determine temporal/spatial regulation of the clustering of specific proteins in response to external stimuli. For example, activation of RTKs signaling pathways determines the phosphorylation of specific tyrosine residues on the receptors and other substrates. It has been shown that SH2 domain-containing proteins, in particular adapter proteins such as Grb2, can drive Liquid-Liquid Phase Separation (LLPS) and protein condensates formation by binding different partners through their PPI domains (two SH3 domains and one SH2 domain in the case of Grb2) [84], thus highlighting a prominent role of protein phosphorylation in such processes. In fact, while Grb2 is recruited through its SH2 domain, additional scaffolding proteins such as Sos1 [85] and Gab2 [86,87] are recognized by Grb2 and provide sites for other specific interactions. Given that tyrosine phosphorylation and protein condensate formation are tightly correlated, and following what has been discussed in previous paragraphs, understanding the molecular basis of the SH2 domain binding mechanism is of primary importance to depict how these domains regulate signal transduction through the productive LLPS, and how their impairment could lead to the disruption of protein condensates.

## 4. The Pathological Role of SH2-Containing Proteins

As mentioned above, SH2-containing proteins are key components of signal transduction pathways, such as RAS-MAPK, JAK/STAT, and PI3K/AKT pathways. Dysregulation of such pathways due to mutations or altered expression of SH2-containing proteins induces several disorders and diseases. Thus, targeting SH2 domains is an interesting therapeutic strategy for drug design and development. In this section, we focus on diseases related to SH2-containing proteins, such as Noonan Syndrome (NS), cancers, and autoimmune diseases.

### 4.1. Noonan Syndrome: A Genetic Disorder Due to Mutations Affecting SH2 Domains from SHP2

Noonan syndrome (NS) (OMIM 163950) is an autosomal dominant disorder characterized by unusual facial features, short stature, and congenital cardiopathies. Other symptoms such as mental retardation, Webbed neck, cryptorchidism, chest deformity, and bleeding diathesis are also commonly associated with this disease. The prevalence of the disease is about 1 in 1000–2500 live births [88,89]. This genetic condition is caused by the hyperactivation of the RAS-MAPK molecular pathway. The mutations of a multi-domain phosphatase called SHP2 (*PTPN11*) protein induce 50% of NS cases.

SHP2 protein is composed of two SH2 domains (N-SH2 and C-SH2) followed by a PTP domain which retains the catalytical activity, and a C-terminal tail [90]. SHP2 possesses a particular mechanism of action, switching between two conformational states. The inactive state (typical of basal conditions) is characterized by a closed autoinhibited conformation in which the N-SH2 DE loop binds to the active site of the PTP domain. Upon interactions with binding partners, the active state adopts an open conformation, with the N-SH2 that moves away from the PTP active site, making it available for substrate recognition and turnover [91,92]. 40 NS-associated SHP2 mutations affecting 30residues close to or part of the N-SH2 domain–PTP domain interface have been described [93]. Mutations of this multi-domain phosphatase protein represent the major cause of Noonan Syndrome (NS) [94]. Currently, there are no effective treatments for such a genetic condition. Thus, understanding the mechanisms underlying SH2-containing-proteins functions on the development of such disease will determine the development of inhibitors of such proteins. 

It is of interest to notice that the majority of the mutations related to the onset of NS occur mostly at the interface between N-SH2 and PTP domains, destabilizing the inactive form of SHP2, provoking the disruption of the autoinhibitory effect and leading to a hyperactivated phosphatase with a consequent gain-of-function (GOF) that enhances the RAS/MAPK pathway activation [93,94]. However, one NS-causing mutation, T42A, is located in the binding pocket of the N-SH2 domain, dramatically increasing enzyme activity [8,95]. Recently our group characterized quantitatively the effect of the T42A mutation on the binding of the isolated N-SH2 domain to a peptide mimicking one of its physiological binders, Gab2 [65]. The interaction between N-SH2 of SHP2 and Gab2 causes the release of its auto-inhibited conformation, triggering signal transduction and being crucial for enzyme activation in the cellular milieu [96]. Kinetic data revealed that the mutation impairs the ability of the N-SH2 domain to release the ligand, consequently promoting the hyperactivation of SHP2 phosphatase activity.

### 4.2. SH2-Containing Proteins and Immunodeficiency Disorders

Severe combined immunodeficiency (SCID) (OMMIM: 600,802) is the name of an ensemble of disorders with several genetic causes. Indeed, SCID is an autosomal recessive disorder caused by the mutations of specific genes. The disease is characterized by severe pulmonary infections, chronic diarrhea, failure to thrive, and persistent candidiasis. The prevalence of such a disorder is about 1 to 58,000 live births [97]. One of the causes is the mutation of the gene encoding ZAP-70. ZAP-70 (Zeta chain-associated protein kinase) is involved in signaling pathways that are important for the development and activation of T cells [74]. ZAP-70 is a 70kDa protein kinase, and it is normally expressed in the proximity of the surface of the membrane of lymphocytes. The protein is constituted of a tandem SH2-domain (N-SH2 and C-SH2), followed by a kinase domain connected to C-SH2 by an SH2-Kinase linker [74]. The pathogenic mutations are located throughout the gene and mostly in the kinase domains. However, as the phosphorylation of Tyr 315 and Tyr319, located in the SH2 kinase linker, is crucial for ZAP-70 activity, their mutations into phenylalanine lead to the inactivation of ZAP-70 downstream signaling events [98]. In addition, the missense mutation P80Q provokes a destabilization of the SH2 domain, leading to a severe SCID [99]. Moreover, mutations of the gene encoding ZAP-70 induce the production of unstable ZAP-70, leading to the abrogation of CD8+ T cell production and inactivation of CD4+ T cells [100]. Consequently, individuals with such mutations are more prone to infections, as their immune system is weakened. 

X-linked agammaglobulinemia (XLA) is a primary immunodeficiency genetic disorder affecting B-lymphocyte development, with an estimated prevalence of 1:200,000 to 379,000 live births and ranked 5th in primary immunodeficiency [101]. In XLA patients, a reduced number of mature circulating B cells can be found, as well as lower serum Ig levels and disrupted lymphoid architecture. XLA is caused by mutations in Bruton’s tyrosine kinase (Btk), involved in B-cell receptor signaling. Around 20% of XLA-causing mutations rest on the SH2 domain and decrease protein stability [102]. Indeed, Btk is constituted of five different protein interacting domains, including SH3, SH2, and kinase domains. Even though the phosphotyrosine sites are located on the SH3 and KD domains, the SH2 domain of Btk has been reported to be critical for phospholipase C-gamma phosphorylation, and mutations of this domain cause XLA [103]. In fact, the main mutations on the SH2 domain of Btk observed in XLA patients are R288Q, R288W, L295P, R307G, R307T, Y334S, Y361C, L369F, and 1370M [103]. Tzeng and colleagues highlighted the inability of SH2 Btk mutants to bind phosphopeptide ligands, which could explain XLA development. In addition, Btk and kinase domain (KD) allosteric interaction possesses a crucial role in kinase activation, showing how XLA mutations in the SH2 domain cause the loss of function phenotype [104]. The absence of this functional protein leads to failure of B-cell development that incapacitates antibody production and subsequently leads to recurrent bacterial infections.

### 4.3. SH2-Containing Proteins and Cancer

**Figure 4:**
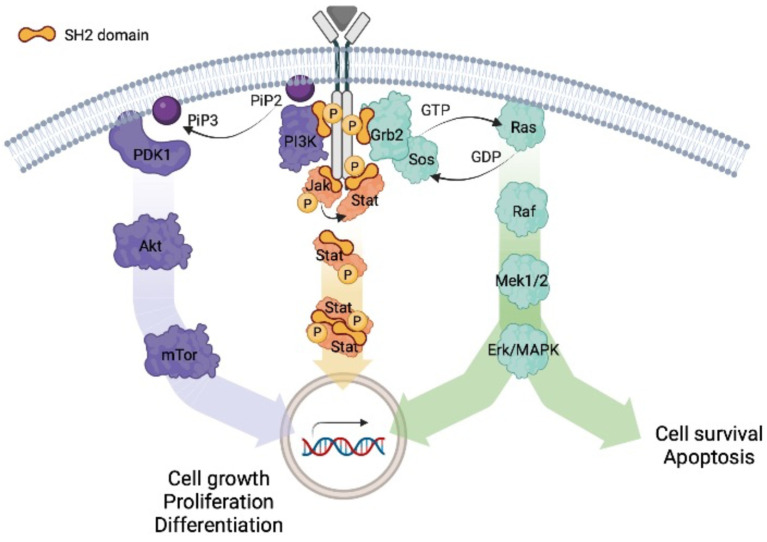
Oncogenic signaling pathways activated by interaction between the SH2 domain of different proteins (Grb2, p85PI3K, Stat). Phosphorylated sites are highlighted.

Several SH2-containing proteins are involved in the development of certain types of cancers, such as breast cancer (Grb2, Grb7, STAT3, and Src), liver cancer (STAT3), Prostate cancer (STAT3, STAP2), Chronic Myelogenous Leukemia, CML (Bcl/Abl). The aberrant activation of signaling pathways (RAS/MAPK, PI3K/AKT, JAK/STAT), including cytokines and growth factors, has a key role in the onset of those types of cancers, promoting metastasis, angiogenesis, and cancer cell division or proliferation. The protein-protein interactions involved in the activation of these pathways are mainly mediated directly by SH2 domains (Figure 4).

Prostate cancer is one of the most common malignancies diagnosed in men (268,490 new cases) [105], causing approximately 350,000 death per year [106]. Prostate cancer progresses in a multi-step fashion, and androgen deprivation therapy is used for advanced and metastatic phases of the disease. MST2 kinase of the Hippo pathway phosphorylates STAT3 via its tyrosine residue located on its SH2 domain [107]. This hinders the SH2 domain of STAT3 from interacting with another phosphorylated counterpart, affecting STAT3 dimerization and activation by IL-6. Thus, mutations occurring on that tyrosine residue increase STAT3 activity and IL-6 expression. Indeed, by investigating the activity of MST2 on STAT3, Tang and colleagues highlighted the effect of the Hippo pathway on prostate cancer through the monomerization of STAT3 [107]. Previous studies carried out by Antonioli and colleagues reported in 2013 that hyperactivation of STAT3 plays a critical role in malignant initiation, tumor progression, and metastatic dissemination [108]. Another SH2-containing protein involved in prostate cancer is the STAP-2 protein [109]. STAP2 is a signal-transducing adaptor protein 2 that promotes prostate cancer growth by enhancing EGFR stabilization [110]. 

Liver cancer is one the deadliest cancers, with approximately 906,000 new cases and 830,000 deaths [111]. Chronic infections with Hepatitis B and C Viruses, HBV and HCV, Cirrhosis, excessive alcohol consumption, and obesity are the key risk factors for disease development. Melvin and colleagues reported that STAT3 affects the progression of liver cancer [112]. The Nemo-like kinase involved in the Ras/MAPK pathway is found to inhibit the phosphorylation of STAT3 by competing with its physiological binder GP130 (via YXXQ motif). GP130 activates STAT3 by recruiting and interacting with the SH2 domain of STAT3 [111], blocking JAK1/STAT3 interaction and consequently dysregulating the downstream events of the JAK/STAT pathway. 

Chronic Myeloid Leukemia (CML) results from the neoplastic transformation originating from hematopoietic stem cells. The incidence of CML is approximately 1.6 per 100,000 population. The initial chronic phase (CP) shows an expansion of granulocytic cells, while the progression to the blast phase (BP) is characterized by a block of cell differentiation resulting in the presence of 30% or more myeloid or lymphoid blast cells in peripheral blood or bone marrow, or extramedullary infiltrates of blast cells. The hallmark genetic abnormality of CML is a t(9;22)(q34;q11) translocation in a ‘Philadelphia chromosome,’ containing a breakpoint cluster region (BCR) sequence, and chromosome 9, containing Abelson proto-oncogene (ABL) sequence. The N-terminal region of c-Abl is replaced in the Bcr-Abl fusion protein by bcr-encoded sequences inducing constitutive activation of Abl with a dysregulated tyrosine kinase activity and a consequent oncogenic ability [113]. The role of the Src homology 2 (SH2) domain of Bcr/Abl in transformation has been extensively studied. The precise SH2/*N*-lobe interaction is required for full activation of c-Abl. In CML cells, active Bcr/Abl phosphorylates BCR’s Y177, recruiting Grb2 through the interaction with the SH2-Grb2 domain [114]. This strong interaction leads to the recruitment of SOS at the myeloid cell membrane determining activation of the Ras/MAPK pathway and then a potent oncogenic proliferation (A schematic representation of the pathway is reported in Figure 4). 

The disease has been correlated to expression levels of SH2 domain-containing protein tyrosine phosphatase-1 (SHP1). This protein is widely expressed in hematopoietic cells assuming a significant role in the regulation of Bcr-Abl, leading to uncontrolled cell proliferation and, additionally, to a reduced expression of the tumor suppressor SHIP1, a human inositol-5-phosphatase [115]. A marked decrease of this protein in CML cell lines and BP compared with CP provides evidence for a significant biological role in disease progression [116]. STAP-1 is a novel Bcr-Abl binding partner, inhibiting the apoptosis of CML stem cells by upregulating BCL-2 and BCL-xL anti-apoptotic genes [117]. This effect is obtained through the activation of STAT5, mediated by the SH3 and SH2 domains of Bcr-Abl [118].

## 5. Pharmacological Strategies Aimed to Regulate/Inhibit SH2 Domains

### 5.1. Peptidomimetics and Small Molecules Inhibitors

Many strategies have been proposed to change the functionality of SH2 domain-containing proteins, such as ABL, BTK, GRB2, SHP2, SRC, and STAT. However, the design of the appropriate interfering molecule must face the concentrated polar charges present in the phosphotyrosine binding pocket, as well as the very well-conserved amino acid sequences in the SH2 domains of different proteins. These aspects required a great effort in designing molecules able to permeate the hydrophobic cell membrane and bind the SH2 domain of a specific protein. Nevertheless, in the last decades, in the search for high-affinity SH2 inhibitors that are specific, stable, and cell-permeable, many different effective compounds have been produced.Historically, the first approaches aimed at blocking the SH2 function were focused on targeting the SH2 phosphotyrosine binding site [119]. Initial compounds tested were the pTyr-mimicking inhibitors (p-Tyr-isosteres), characterized by the presence of a phosphorylated Tyr with the phosphate bridging oxygen replaced by a methylene unit, thus ensuring stability to hydrolysis by phosphatases [120]. Many peptidomimetics-based inhibitors have been developed containing a phosphonomethyl phenylalanine residue (Pmp). For example, a hydroxy benzyl phosphinate has been developed, displaying a better cell permeability than other Pmp peptidomimetics due to its reduced negative charges and binding with relatively high affinity (K_D_ of 0.53 μM) toward the SH2 domain of Grb2 protein [121,122]. More recently, peptidomimetics, with enhanced cell penetration, containing 4-phosphonodifluoromethylcinnamate have been developed by masking the phosphonate hydroxyls with reversible pivaloyloxymethyl protecting groups. A derivative of these compounds has been found to have a very high affinity to STAT3 SH2 (IC_50_ of 162 nM), and to inhibit the STAT3 phosphorylation, essential for its dimerization-dependent function(s), at 100 nM in MDA-MB-468 breast cancer cell line [123]. Moreover, the compound showed a very high grade of selectivity for STAT3 SH2, not inhibiting PI3K, Src SH2, and poorly binding to other STAT SH2 domains [123,124,125]. This result was mainly achieved thanks to the mimic amino acids sequence present in the compound, in which the auxiliary specificity elements (i.e., glutamine mimics) can be placed in the binding pocket of the STAT3 SH2 domain but not in those of the other SH2-containing proteins [124].

A different strategy based on peptidomimetics was employed for Syk kinase (ZAP-70). In this protein, the simultaneous binding of the two Syk SH2 domains to a dually phosphorylated receptor triggers allosteric rearrangement and activation of the kinase [126]. Thus, peptidomimetic inhibitors were developed to contain two distinct phosphorylated binding regions, separated by a flexible linker which acquired a pivotal role in assuring binding efficiency to the two Syk SH2 domains [127]. With this aim, photo-switchable rigid linkers were explored to assess the effects of cis-trans isomerization on binding affinity. By incorporating a well-studied photo-switchable core, 4-aminomethyl phenylazobenzoic acid (AMPB), as a linker between the two phosphorylated regions, it has been possible to achieve a compound with K_D_ = 65 nM for the trans isomer obtained with visible light irradiation, and K_D_ = 860 nM for cis isomer obtained with irradiation at 366 nm [128]. Even with still some drawbacks that need to be solved [129], photo-switchable systems could represent a novel strategy for developing inducible inhibitors.

Another interesting approach has been focused on the research of constrained peptides (bicyclic) that mimic pTyr using discontinuous epitope-binding surfaces and that aim to overcome the main problem of peptides and peptidomimetics, linked to poor cytosolic absorption by removing the charged phosphate group (non-phosphate peptide) with a carboxylate group [130]. These efforts have led to the development of the BC1 compound, a bicyclic peptide able to inhibit Grb2-SH2 with an IC_50_ of 350 nM. Unfortunately, even if BC1 demonstrated good cellular uptake in breast cancer cells, no antiproliferative effect was observed. This compound could bind anti-pTyr antibodies, demonstrating that a carboxylate group, instead of phosphate, could successfully mimic pTyr [131,132].

Peptide and peptidomimetics often performed poorly in cell-based assays, mainly due to poor cytosolic penetration or the absence of biological activity. The development of small molecule inhibitors might overcome these issues, possibly acting as pTyr-mimics but with better cytosolic penetration. One of the most promising classes of compounds as small molecule pTyr isostere seems to be the sulfonamide class [133]. A compound found after a screening of 920,000 molecules and then further improved (C188-9) was tested as a small molecule inhibitor of the STAT3 SH2 domain [134]. The C188-9 molecule showed a K_D_ of 4.7 nM, and its treatment (100 mg/kg/day) in mouse xenograft models with UM-SCC-17B head and neck squamous cell carcinoma determines a significant reduction in tumor size [134]. Additionally, under the new name of TTI-101, it entered the phase I clinical trial for Tvardi Therapeutics.

### 5.2. Allosteric Inhibitors

Unfortunately, so far, very few of the strategies above-described produced compounds that entered clinical trials or have effective results in limiting or influencing the progression of pathologies without excessive toxicity (off-targets). For these reasons, new pharmacological strategies have been developed to avoid all the problems-related pitfalls in targeting the SH2 p-Tyr binding site. One of the most promising is focused on targeting the SH2 domain allosterically.

There are several protein systems in which SH2 domain(s) act as a regulator of the activity of an entire protein, often determining the switching from inactive to active conformation [104,126,135]. An interesting and well-studied example is represented by the SHP2 protein, which plays a central role in mediating signal transduction downstream of receptor tyrosine kinases (RTKs) such as EGFR, ERBB2, c-MET, and FLT3) or FRS2 protein (STAP) [136]. Considering the SHP2 structural and functional properties (described in paragraph 4.1), the possibility of pharmacologically stabilizing the autoinhibited conformation to block the cellular signaling became an attractive approach to fighting several types of cancers [136] or syndromes (e.g., Noonan syndrome) [95]. Indeed, these pathologies are often characterized by the presence of activating mutations of SHP2 (e.g., E76Q, F285S, S502P, D61V, E76K) mainly located at the interface between N-SH2 and PTP domain, and able to abolish the inhibitory interaction between its N-SH2 domain and phosphatase domain. For this reason, there were extensive efforts in searching for compounds able to target this interface to stabilize the autoinhibited conformation.

One of these inhibitors, SHP099, was found after a screening and improvement of a library of 100,000 compounds [137]. SHP099 is a small compound belonging to the class of pyrazinyl molecules. The crystal structure of SHP099 in complex with SHP2, at 1.7Å resolution, revealed that SHP2 was in an auto-inhibited, inactive conformation with the N-terminal SH2 domain blocking the active site, bound to the central tunnel formed at the interface of the N-SH2, C-SH2, and PTP domains. Key interactions included Hydrogen bonds involving residues Arg111 and Phe113 were of key importance. Those residues are physically located on the linker between the N-SH2 and C-SH2 domains, as well as Glu250 from the PTP domain. The dichlorophenyl group of SHP099 explored hydrophobic interactions with Leu254, Gln257, Pro491, and Gln495 side chains of the PTP domain. No activity was reported versus SHP1, the closest homolog of SHP2, sharing 61% amino acid sequence identity, supporting its high degree of target selectivity. The selectivity of SHP099 for SHP2 over SHP1 might be due to the repositioning of the linker between the two SH2 domains in the homolog SHP1. This change would remove key SHP099 interactions (with Arg109 in SHP1 and Arg111 in SHP2) and yield a significantly larger central tunnel, unfavorable for an SHP099 effect. SHP099 was reported to be a highly potent (IC_50_ = 0.071 μM) SHP2 inhibitor. It suppressed the RAS-ERK signaling with an IC_50_ of about 25 μM and inhibited the proliferation of receptor-tyrosine-kinase-driven human hematopoietic or colon rectal cancer cells in vitro. It was shown to be efficacious in controlling tumor growth in mouse tumor xenograft models [137]. In addition, evidence has been reported on the efficacy of SHP009 to inhibit the mutant E76A SHP2, the form frequently identified in Noonan syndrome and leukemia, for which an IC_50_ of 0.12 μM has been measured [138].

In search of optimization of the pyrazine class of allosteric SHP2 inhibitors, TNO155 has been synthesized. This molecule, strictly related to SHP099, is a potent inhibitor of SHP2 (IC_50_ = 0.011 μM), with high cellular potency (p-ERK IC_50_ = 0.011 μM; antiproliferation of esophageal cancer cells KYSE-520 IC_50_ = 0.100 μM) and high solubility (0.736 mM). It can bind to the tunnel site of SHP2, although with interactions different from those reported for SNP99. Antitumor effects could also be recorded in the EGFR-driven esophageal carcinoma xenograft model, KYSE-520, at a dose lower than those of SNP099; as SNP099, it did not cause body weight loss, but differently from SNP099, it proved negative in vitro phototoxicity tests [139]. TNO155 has recently entered phase I clinical trial for advanced solid tumors both as a single agent and in combination therapies with narzatinib (inhibitor of the tyrosine kinase activity of the EGFR bearing activating mutations such as Tyr790Met), ribociclib, (selective inhibitor of CDKs 4and 6) or spartalizumab (humanized monoclonal antibody against PD-1) [140].

Another SHP2 allosteric inhibitor molecule, LY6, was found by exploiting a combination of computational drug design and experimental assay. LY6 was rationale-designed to bind an alternative pocket present between C-SH2 and PTP domain and, similarly to SHP099, to block SHP2 in the inactive state. Even if the co-crystal structure of this compound with SHP2 is still lacking, structure–activity relationship analyses of optimized derivatives suggest that the main binding mode is characterized by strictly connecting the C-SH2 and PTP domain. Additionally, despite LY6 inhibiting wild-type SHP2 and SPH2 E76K with low affinity (IC_50_ 9 μM and 7.7 μM, respectively), it seems to be highly selective for SHP2 rather than SHP1 (about 7-fold). In Ba/F3, an IL-3-dependent murine pro-B lymphoma cell line, LY6 suppressed cell proliferation and inhibited the IL-3-induced phosphorylation of Erk, Akt, Jak2, and Stat5. Also, mouse and patients cells with Juvenile Myelomonocytic Leukemia (JMML) bearing E76K SHP2 are more sensitive to LY6 than wild-type cells, highlighting its possible use for the treatment of PTPN11-associated malignancies [141].

The possibility for dual allosteric inhibition of SHP2 has also been evaluated, considering that additional allosteric binding sites have been predicted on both sides of the N-SH2/PTP domain interface, i.e., the “latch” binding site, located approximately 20 Å from the tunnel, and the “groove” site on the opposite side of the SHP2 protein. The triazolo-quinazolinone compounds SHP244, 844, and 504 have been reported to bind the latch binding site and inhibit the activity of near full-length SHP21-525 protein with IC_50_ values of 60, 18.9, and 21 µM, respectively. X-ray structure analysis of SHP2 with SHP099, bound at the tunnel site, and SHP244, 844, and 504, at the latch binders, showed that each of the three triazolo-quinazolinone compounds bound to SHP2 simultaneously with SHP099. Although their efficacy was much lower than those of SHP099 (IC_50_ 0.070 μM), co-treatment of KYSE-520 cells with SHP099 (0.2 μM) and SHP504 (30 μM) has been shown to result in a stronger downregulation of DUSP6, a pharmacodynamic marker of the MAPK signal downstream of SHP2, with respect to the effect, compared to each of the single agents, thus paving the way to new possible combined approaches [142].

To summarize, a list of effective molecules on the SH2 domain binding is reported below, in Table 3.

## 6. Conclusions

SH2 domains are prototypical “readers” of phosphorylated tyrosine residues. Since tyrosine phosphorylation is a post-translational modification that regulates several physiological and molecular pathways in the eukaryotic cell, SH2 domains represent a fundamental piece in the intricate puzzle of cell signaling. As a consequence, dysregulation of SH2-domains mediated PPIs are rigorously involved in the onset of several pathologies. In this review paper, we summarized the current knowledge about the folding and binding properties of SH2 domains, as well as their roles played in pathogenesis. A great effort has been made over the years to understand the determinants of the function of SH2 domains in terms of binding mechanisms with their ligands and regulation of SH2 domain-mediated molecular pathways. However, while through structural and biophysical approaches, we could understand the role of residues located in loops and binding pockets in determining affinity and specificity for their ligands, we still have to investigate the role of single residues in the early events of the association and late events of complex stabilization of the binding event. This could represent a fundamental step toward a better comprehension of the effect of pathological mutations on the binding of SH2 domains and, consequently, to the design of pharmaceutical strategies aimed at regulating their (mis)functions. In particular, a synergistic approach based on site-directed mutagenesis, binding experiments, and molecular dynamic simulations (which could take advantage of a large amount of structural information available about SH2 domains) it would be possible to map, at an almost atomic level, the contribution of single residue side-chains on the binding of the inhibiting molecules, providing useful insights toward the design of inhibitor molecules displaying increased affinity and performance. Our analysis leads to the evidence that the intense pharmacological interest in SH2 domains is of fundamental importance to developing new therapeutics and that further analysis of their binding mechanisms and the determinants of specificity is demanded to improve our ability to inhibit/regulate SH2-mediated protein-protein interactions.

## Figures and Tables

**Table 1:** A comprehensive list of human SH2-domain-containing proteins. For each protein listed, the number of SH2 domains, the function, and the Uniprot Entry number is reported.

Family	Human Genes	Protein Short Names	SH2 Domains	Molecular Functions	Uniprot Entry
ABL	ABL1, ABL2	Abl-1, Abl-2	1	Enzyme (Tyrosine-kinase)	P00519, P42684
CBL	CBL, CBLB, CBLC	Cbl, Cbl-b, Cbl-c	1	Enzyme (E3 ubiquitin-protein ligase)	P22681, Q13191, Q9ULV8
CHN	CHN1, CHN2	Chin-1, Chin-2	1	Regulator (GTPase activity activator)	P15882, P52757
CRK	CRK, CRKL	Crk, Crkl	1	Adaptor protein	P46108, P46109
CSK	CSK, MATK	Csk; Ctk	1	Enzyme (Tyrosine-kinase)	P41240, P42679
DAPP1	DAPP1	Dapp-1	1	Adaptor protein	Q9UN19
FPS	FPS (FES), FER	Fps (Fes), Fer	1	Enzyme (Tyrosine-kinase)	P07332, P16591
FRK	FRK, BRK, SRMS	Frk, Brk-1, Srms	1	Enzyme (Tyrosine-kinase)	P42685, Q8WUW1, Q9H3Y6
GRB	GRB2, GADS, GRAP	Grb2, Gads, Grap	1	Adaptor protein	P62993, O75791, Q13588
GRB7	GRB7, GRB10, GRB14	Grb7, Grb10, Grb14	1	Adaptor protein	Q14451, P0CE43, Q14449
JAK	TYK2, JAK1, JAK2, JAK3	Tyk2, Jak1, Jak2, Jak3	1, atypical	Enzyme (Tyrosine-kinase)	P29597, P23458, O60674, P52333
NCK	NCK1, NCK2	Nck1, Nck2	1	Adaptor protein	P16333, O43639
PI3KR	PIK3R1, PIK3R2, PIK3R3	p85A, p85B, p55G	2	Enzyme (1-phosphatidylinositol-3-kinase)	P27986, O00459, Q92569
PLCg	PLCG1, PLCG2	Plcg1, Plcg2	2	Enzyme (phosphatidylinositol phospholipase)	P19174, P16885,
PTPN	PTPN6, PTPN11	Shp2 (Ptp)	2	Enzyme (Tyrosine phosphatase)	Q06124
RASA1	RASA1	Gap (RasGap, p120RasGap)	2	Regulator (GTPase activity inhibitor)	P20936
RIN	RIN1, RIN2, RIN3	Rin1, Rin2, Rin3	1	Regulator (Ras effector)	Q13671, Q8WYP3, Q8TB24
SH2B	APS, LNK, SH2B	Aps, Lnk, Sh2b	1	Adaptor protein	O14492, Q9UQQ2, Q9NRF2
SH2D1	SH2D1A, SH2D1B	Sh2d1A (Sap), Sh2d1B	1	Adaptor protein	O60880, O14796
SH2D2	SH2D2A, HSH2D, SH2D7	Sh2d2a, Hsh2d, Sh2d7	1	Adaptor protein	Q9NP31, Q96JZ2, A6NKC9
SH2D3	SH2D3A, SH2D3C, BCAR3	Sh2d3a, Sh2d3c, Bcar3	1	Adaptor protein	Q9BRG2, Q9QZS8, Q9QZK2
SH2D4	SH2D4A, SH2D4B	Sh2d4a, Sh2d4b	1	Adaptor protein	Q9H788, Q5SQS7
SH2D5	SH2D5	Sh2d5	1	Adaptor protein	Q6ZV89
SH3BP2	SH3BP2	Sh3bp2 (3bp-2)	1	Docking protein	P78314
SHB	SHB, SHD, SHE, SHF	Shb, Shd, She, Shf	1	Docking protein	Q15464, Q96IW2, Q5VZ18, Q7M4L6
SHC	SHC1, SHC2, SHC3, SHC4	Shc1, Shc2, Shc3, Shc4	1	Docking protein	P29353, P98077, Q92529, Q6S5L8
SHIP	SHIP1, SHIP2	Ship-1, Ship-2	1	Enzyme (Phosphatidylinositol (PtdIns) phosphatase)	P97573, O15357
SLAP	SLAP, SLAP2	Slap-1, Slap-2	1	Adaptor protein	Q13239, Q9H6Q3
SLP76	BLNK, LCP2, CLNK, SCIMP	Slnk, Blnk, Slp76, Mist, Slp65/Slp76	1	Adaptor protein	Q8WV28, Q13094, Q7Z7G1, Q6UWF3
SOCS	SOCS1, SOCS2, SOCS3, SOCS4, SOCS5, SOCS6, SOCS7, CISH	Socs1, Socs2, Socs3, Socs4, Socs5, Socs6, Socs7, Cish	1	Enzyme (protein ubiquitination)	O15524, O35717, O14543, Q8WXH5, O75159, O14544, O14512, Q9NSE2
SRC	SRC, FYN, LCK, FGR, YES, LYN, HCK, BLK	Src, Fyn, Lck, Fgr, Yes, Lyn, Hck, Blk	1	Enzyme (Tyrosine-kinase)	P12931, P06241, P06239, P09769, P07947, P07948, P08631, P51451
STAP	BRDG1, BKS	Brdg1 (Stap-1), Bks (Stap-2)	1	Docking protein	Q9ULZ2, Q9UGK3
STAT	STAT1, STAT2, STAT3, STAT4, STAT5, STAT5B, STAT6	Stat1, Stat2, Stat3, Stat4, Stat5, Stat5b, Stat6	1	Transcription factor	P42224, P52630, P40763, Q14765, P42229, P51692, P42226
SPT6	SUPT6H	Supt6h (Spt6)	1	Transcription factor	P42226
SYK	ZAP70, SYK	Zap-70, p72-syk	2	Enzyme (Tyrosine-kinase)	P43403, P43405
TEC	BMX, TEC, BTK, ITK, TXK	Bmx, Tec, Btk, Itk, Txk	1	Enzyme (Tyrosine-kinase)	P51813, P42680, Q06187, Q08881, P42681
TNS	TNS1, TENS2, TNS3, TNS4	Tensin-1, Tensin-2, Tensin-3, Tensin-4	1	Cytoskeletal protein	Q63HR2, Q63HR2, Q68CZ2, Q8IZW8
VAV	VAV1, VAV2, VAV3	Vav1, Vav2, Vav3	1	Regulator (Guanine-nucleotide releasing factor)	P15498, P52735, Q9UKW4

**Table 2:** List of SH2 domains whose structures are currently available (adapted and updated from [53]). The abbreviation pY indicates the phosphotyrosine, σ an acidic residue, x an undefined residue, Ψ a hydrophobic residue, and n/a not available information.

Group		SH2 Domain	βD5	Motif	Peptide Specificity Residues
**I**	**IA**	SRC, FYN, FRK, LCK, HCK, BLK, ABL1, ABL2, ITK, BTK, TXK, ZAP70_N & C, SYK_N &C, NCK1, NCK2, BRK, YES, LYN	Y/F	pY-σ-σ-ψ	+3
	**IB**	SAP, EAT2, SHIP1, SHIP2, CRK, CRKL, RasGAP_C, CHK, BRK	Y/F	pY-x-x-ψ	+3
	**IC**	GRB2, GADS, GRB7, GRB10, GRB14, ALX, FES, BMX, CSK, SH3BP2, HSH2D, TENC1, FER, SRM	Y/F	pY-x-N	+2
	**IE**	α-Chimaerin, β-Chimaerin	Y/F	n/a	?
**II**	**IIA**	VAV, VAV2, PI3K-p85α_N & C, PLC-γ1_C, PLC-γ2_N & C, SHP-1_N & C, SHP2_N & C, SOCS2 & 3 & 4 & 6	I/C/L/V/A/T	pY-ψ-x-ψ	+3
	**IIB**	APS, SHB2, SHC1, BLNK	L/I	pY-[E/D/x]-x-ψ	+3
	**IIC**	BRDG1, BKS, CBL	Y/T	pY-x-x-x-ψ	+4
	**IID**	SPT6	V	pY-[P/I]-P-[K/R]-M	?
**III**	**III**	STAT1 & 3 & 5A	V/L	pY-x-x-Q	+3

**Table 3:** List of effective SH2-domain inhibitors (* Interactions with different amino acid residues).

Inhibitors Classes	Active Moiety or Binding Site	Inhibitor Name	Targeted Protein	K_D_° or IC_50_#	References
Peptidomimetis					
pTyr-isosteres	Phosphonomethyl-Phe	Pmp	PI 3-kinase p85	0.01–0.02 μM°	[120]
	Hydroxy benzyl phosphinate		Grb2	0.53 μM °	[121,122]
	4-phosphonodifluoromethylcinnamate		Stat3	0.16 μM #	[124,125]
	Sulfonamides	C188-9	Stat3		[133,134]
		(TTI-101)			
Photo-switchable linkers	4-aminomethyl phenylazobenzoic acid		Zap-70	0.065–0.8 μM °	[126,127,128,129]
Bicyclic peptides	Carboxylate group	BC1	Grb2	0.35 μM #	[130,131,132]
Allosteric inhibitors					
Pyrazines	N-SH2/C-SH2/PTP interface	SHP099 *	Shp2	0.071 μM #	[137,138]
	N-SH2/C-SH2/PTP interface	TNO155 *	Shp2	0.011 μM #	[139,140]
	C-SH2/PTP interface	LY6	Shp2	9 μM #	[141]
Triazolo-quinazolinones	“latch” N-SH2/PTP binding site	SHP244	Shp2	60 μM #	[142]
	“latch” N-SH2/PTP binding site	SHP504	Shp2	21 μM #	[142]
	“latch” N-SH2/PTP binding site	SHP844	Shp2	18.9 μM #	[142]

## Data Availability

No new data were created or analyzed in this study. Data sharing is not applicable to this article.
